# Irregularities of the Teeth and Their Surgical Treatment

**Published:** 1881-01

**Authors:** Francis Fox


### ARTICLE IV.

### *Irregularities of the Teeth and their Surgical Treatment.*

BY FRANCIS FOX.

Early decay of the teeth which marks the present generation is due, the author thought, to malnutrition occurring in the earlier periods of life. At about seven months after birth a process of absorption is set up in the walls of the crypt and parts superimposed, and by this process the crowns of the temporary teeth become visible above the surface of the gums. When the crowns of the teeth have erupted this absorptive action for a time ceases; and a renewal of the developmental process ensues, by which the alveoli are built up around the roots of the teeth. At about four years of age the temporary dentition is perfected, and soon after this perfection is reached absorption again sets in, commencing now in the roots of the teeth, and these, together with their alveola processes, are gradually removed, their permanent successors replacing them by a similar process of absorption of crypt and development of alveolar structures.

The important point to bear in mind is the fact that the alveolar portion of the jaws is developed with each dentition, so that a previous alveolar structure can have little to do with the position of the succeeding teeth, except as it may present an obstacle to their onward progress in consequence of its non-absorption. As to the development of the jaw bones, these bones consist of two portions, (1) an alveolar structure, developed with the temporary teeth, absorbed with them, and again re-developed with the permanent teeth; and (2) a basal portion. This base is more prominently marked in the lower jaw, in which the inferior dental canal very emphatically indicates the junction between the two portions of the bone. The base of the jaw when once formed remains in pretty much the same condition throughout life, except in advanced old age, when the muscles of mastication are no longer in full use, and then in a slight degree it becomes wasted. In the superior maxillæ at birth the alveolar processes descend but little below the level of the palatial plates, and the anterior and posterior parts are but little developed. As age advances the alveoli lengthens, the tuberosities increase in size, and an active development of bone takes place in these situations. The tuberosities are to the upper maxillæ what the coronoid processes are to the lower jaw. From these points the alveolar line is lengthened. In the lower jaw an alteration in the position of its articular surfaces and ascending rami, together with an absorption of the coronoid processes, accompanies the development of the posterior permanent teeth. The jaws elongates by additions to its posterior cornua. The capacity of the jaws in childhood is nearly equal to the anterior portions of the adult bones; for the ten anterior teeth of the permanent set in each jaw replace the temporary, and occupy the same position as these, so that this part of the jaw in adult life is pretty much the same as in childhood. If contracted then it will remain so throughout life, and no subsequent development in the posterion regions will tend to explain it. The replacement of the temporary teeth by their successors is effected by a purely physiological process, and is absolutely independent of pressure.

There seems to be a physiological law by which the cells composing the absorbent papilla in the neighborhood of a developing tissue have the power of absorbing a mature structure. That pressure has nothing to do with the process may be proven by the fact, that in cases where the shedding of the first teeth has taken place prematurely, a layer of bone has been observed to intervene between the crown of the advancing tooth and the base of the socket of its predecessor. At the time when the temporal teeth are about to be shed, in the well-developed jaw a decided separation between contiguous teeth is noticeable; and this circumstance is a fair indication of future regularity in the succeeding dentition, and a proof that this portion of the jaw has already been prepared to receive the larger permanent teeth. If the process of absorption continues uninterruptedly, the roots of the temporary teeth will be gradually removed, leaving little more than the shells of the crowns, which readily drop from the gum as their successors are in turn ready to occupy their places. But should any arrest in this process occur (and such is far from an uncommon circumstance,) these temporary organs are liable to offer considerable obstacles to the regular advance of their permanent successors.

The causes of irregularity in the position of the teeth may arise during the developmental periods of life, and are then due to a want of proportion in the size of the teeth and jaws, or to a faulty development of the jaw bones; or the displacement may depend on some accidental circumstances arising subsequently, such as the prolonged retention of the temporary teeth, the presence of supernumerary teeth, the habit of "thumb sucking," or the undue pressure from an hypertrophied tongue. There is abundant evidence to prove how frequently such deformity depends upon hereditary influences. The conditions of life to which our race has for many generations been subjected seems to have lessoned the necessity for the broad and well-formed jaws which were so characteristic of our ancestors, and for many years the advances in civilization have been marked by a deterioration in the capacity of our jaw-bones. Mr. Coleman, in some interesting investigations made several years ago, found that the percentage of contracted jaws was immeasurably greater in the children of the well-bred population than in those of less refined cultivation. The prolonged retention of temporary teeth is frequently associated with irregularity in their successors, and is probably often the cause of such irregularity. The presence of supernumerary teeth in the dental arch may prevent the normal members from assuming their proper places; but doubtless a disproportion of size between the teeth and jaws is of all causes of irregularity the most common. This disparity leads to a crowding of the teeth, sometimes to such an extent as to altogether prevent the eruption of some one or more of the dental series, such remaining impacted in the substance of the jaws. Certain injuries in early life may occasion displacement of the teeth, especially in the lower maxilla, such as the contraction of cicatrices about the face and neck. Mr. Salter, in his work on Dental Pathology and Surgery, treats the subject of irregularities of the teeth under two heads—(*a*) simple regularity, in which the misplacement is confined to one jaw, and is independent of the position of the teeth in the opposite jaw; (*b*) compound irregularity, which depends upon the position of the teeth in the opposing jaw.

In "simple irregularity"—that is, where the misplacement is confined to one jaw—the crown only of the tooth may be irregularly placed, the apex of the root retaining its normal position; or the entire tooth may be displaced, or faulty in its development. Such irregular teeth are often entirely removed from the dental arch, and may be impacted in the substance of the jaw-bones. In the former condition, when the apex of the root retains its normal position, much good may be effected by judicious treatment, but in the latter case little can be done to remedy the evil, except by the removal of the displaced tooth. As examples of "simple irregularity," we may mention the appearance of the upper canines above the alveolar ridge, or in the palate, owing to insufficient room for them in the dental arch. An early loss of their predecessors, by permitting the first bicuspid and the lateral incisors to approach each other, is not unfrequently the immediate cause of this displacement. Sometimes, however, the retention of the temporary canine, or the presence of a supernumerary tooth, will occasion its deformity.

An overlapping of the incisors is another form of "simple irregularity," and frequently requires for its treatment a resort to some mechanical appliance in order to obtain regularity in the position of these teeth. Another not uncommon form of irregularity is where an incisor tooth is more or less twisted, sometimes to such a degree that the side of the crown will occupy the position of its anterior surface. A forcible twisting of the tooth into its right position is very generally adopted. Some, however, are averse to this prompt treatment, and suggest the employment of a plate carefully adjusted to the palate, and having certain properly constructed points of resistance. An unsightly separation of the central incisors in the upper jaw sometimes occurs, and the teeth may be readily drawn together, but have a great tendency to return to their former position.

In treating these cases, great care should be taken to prevent the ligature from slipping below the edge of the gum—between the necks of the teeth and the gum—for the irritation set up by such a mishap has been known to cause the death of the tooth. In order to prevent this displacement of the ligature, a small vulcanite plate may be constructed, to which the ligature can be attached, and thus prevented from shifting its position. The second form of irregularity of the teeth—that depending upon the position of the teeth in the opposing jaw—is much more complicated. As an example, might be cited the "underhung jaw," in which the "bite" is intersecting; some, or all of the six front upper teeth being shut behind the corresponding teeth in the lower jaw. This condition, in its extreme extent, arises from an undue development of the lower over the upper jaw, or from a want of development in the superior maxillary bones. It may also arise from a retardation in the eruption of the superior incisors, or by these teeth being pushed inwards by the prolonged occupation of the dental arch by their temporary predecessors. An early treatment of this irregularity is all important, and should consist in preventing the contact of the opposing teeth.

An opposite condition of the lower jaw sometimes occurs, in which the lower incisor teeth bite close up to the palate, so that they press against the necks of the upper teeth, and push them forward. A separation of the teeth in the anterior portions of the jaws has been described, and is occasioned by a congenital malformation of the lower jaw. The early obliquity in the position of the ascending rami is unduly maintained, and there is a want of development in the alveolar portions of the jaw, especially in the regions of the molar teeth. This irregularity may be caused by the contraction of a cicatrix in the throat or neck. The bicuspid teeth are not infrequently misplaced, and, when so, they usually occupy a too inward position. This may arise solely from their having been prevented from assuming their proper positions in the dental arch by the prolonged retention of the temporary molars. But usually it is dependent upon a diminished capacity of the jaw, and in the upper jaw is generally associated with a projection of the incisors, and a more or less elevation of the palate constituting the V-shaped jaw, or "rabbit-mouth." This malformation is congenital, but, except in very exaggerated cases, is not very manifest until the posterior permanent teeth are about to be erupted, when the additions to the superior maxilla have been made in the posterior regions.

The newly-formed bone, which has been gradually developing, is now found to be placed at an angle with the pre-existing alveolar line. This abnormal development has arisen in order to effect an harmonious arrangement with the other bones of the cranium. The maxillary bones having been imperfectly developed during early childhood their posterior borders not being sufficiently divergent, the subsequent additions for adult confirmation are placed in a wider circle; hence the point of junction between the two parts (the old and the new, so to speak,) is marked by an angle of more or less extent. It is usually associated with great delicacy of constitution, and may occur in those of weak mental powers, but is often observed in persons of great intellectual capacity. The treatment of these cases consists in endeavoring to gain increased space in the dental arch, and to diminish the projection of the upper front teeth; but is, as a rule, more or less unsatisfactory. Irregularity in the wisdom teeth is sometimes met with, and may occasion most serious mischief, when extraction is the remedy. Transposition of the teeth is rare, and is usually met with anteriorly; and also inversion, which is still rarer.—*London Lancet.*

